# Supplementary material for: Human PrP E219K: a new and promising substrate for robust RT-QuIC amplification of human prions with potential for strain discrimination
Source: Microbiol Spectr. 2025 Jul 10;13(8):e00292-25. doi: 10.1128/spectrum.00292-25 (PMC12323364; doi:10.1128/spectrum.00292-25)
Supplement: Supplemental figures — Figures S1 to S3. [file spectrum.00292-25-s0002.pdf]

Supplementary Figure Legends

Supplementary Figure 1: western-blot analysis of PrP^{res} content in brain homogenates from tg650 mice (A) and human patients (B) infected with sCJD or vCJD prions at the terminal stage of the disease. A) Lane 1: molecular weight marker (MM); lanes 2 to 7: purified recombinant human PrP (25 to 0.7 ng); lanes 8 to 13: tg650-sCJDs samples; lanes 14 and 15: tg650-vCJD samples. B) Lanes 1 and 17: molecular weight marker (MM); lanes 2 to 7: purified recombinant human PrP (25 to 0.5 ng); lanes 8 to 13: human samples; lanes 14 to 16: human vCJD samples. For all samples, the amount of brain loaded per lane is expressed as "brain equivalent" (mg), *i.e.* the calculated mass of original processed tissue in the sample volume.

Supplementary Figure 2: RT-QuIC amplification reactions seeded with tg650 mouse brain homogenates. A to G) tg650-MM1, tg650-MV1, tg650-VV1, tg650-MM2-c, tg650-MV2, tg650-VV2, tg650-vCJD, respectively and H) Uninfected tg650 brain homogenate. Data represent the mean ± SD of fitted positive amplification reactions across serial dilutions.

Supplementary Figure 3: RT-QuIC amplification reactions seeded with human patient brain homogenates. A to G) MM1-sCJD, MV1-sCJD, VV1-sCJD, MM2c-sCJD, MV2-sCJD, VV2-sCJD, vCJD, respectively and H) Uninfected human brain homogenate. Data represent the mean ± SD of fitted positive amplification reactions across serial dilutions.

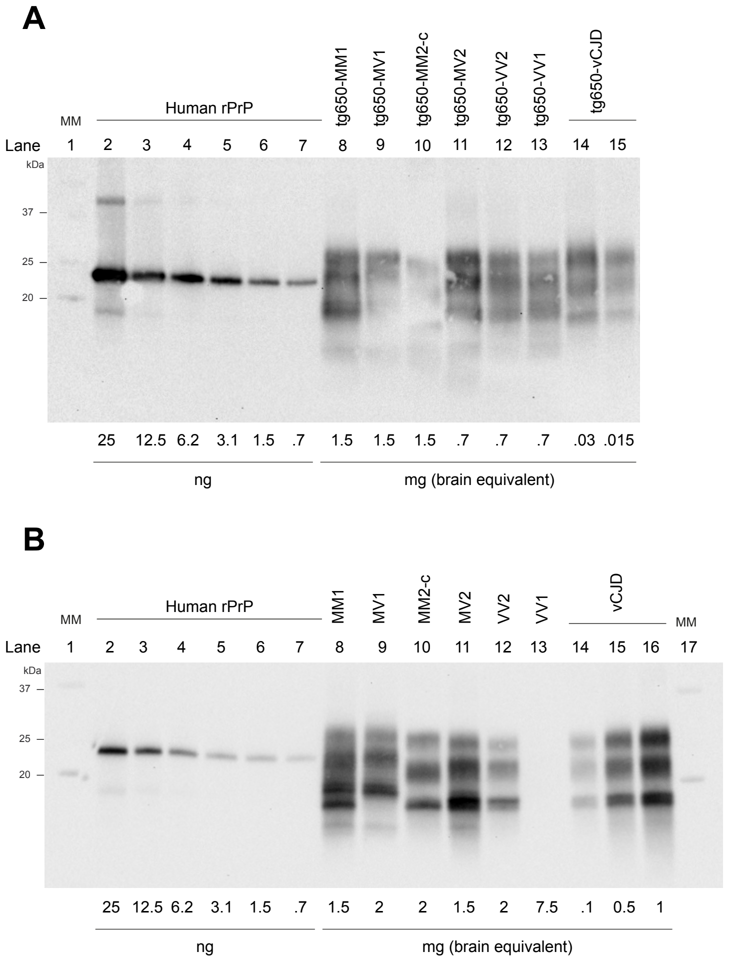

Supplementary Figure 1

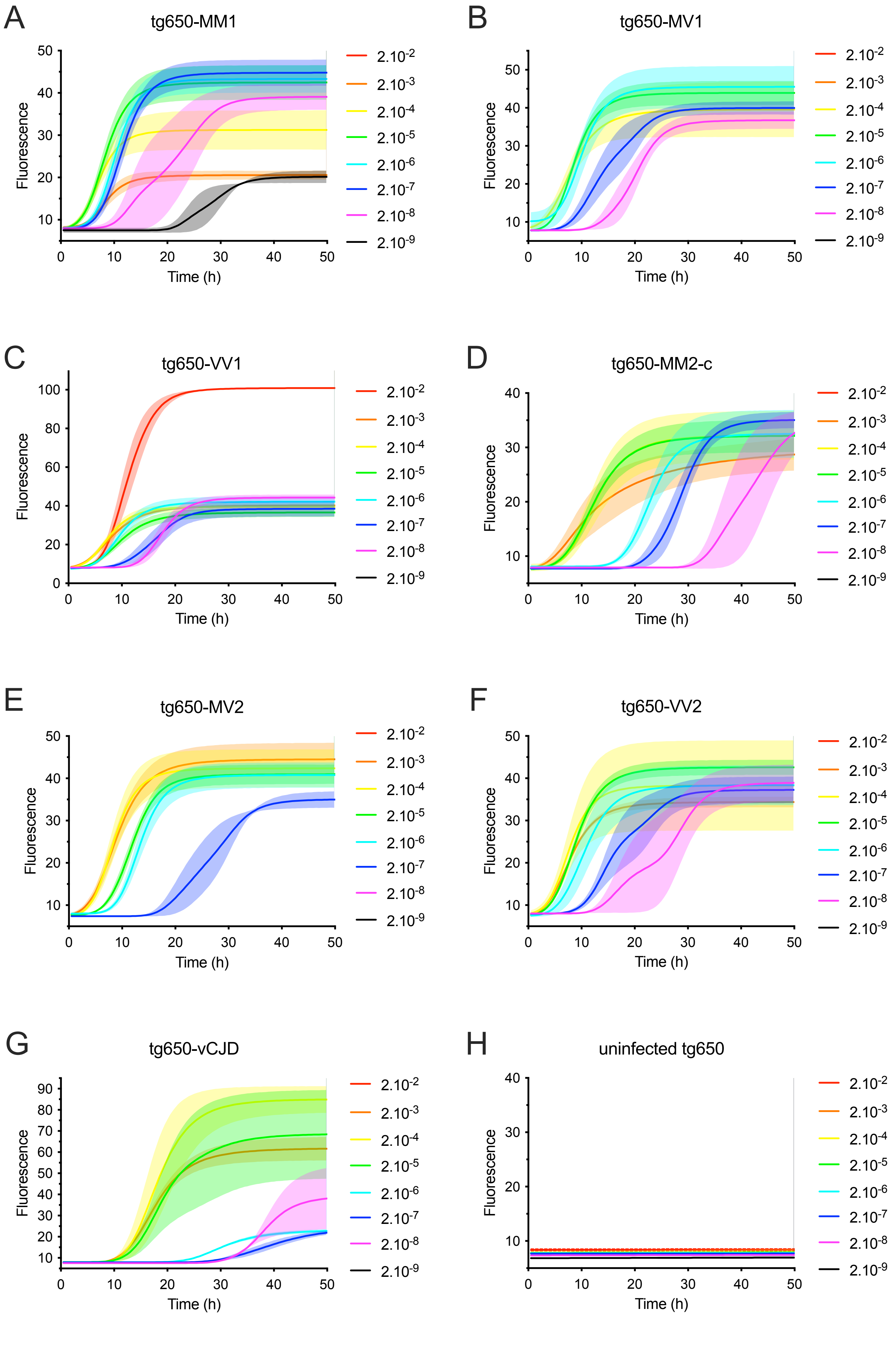

Supplementary Figure 2

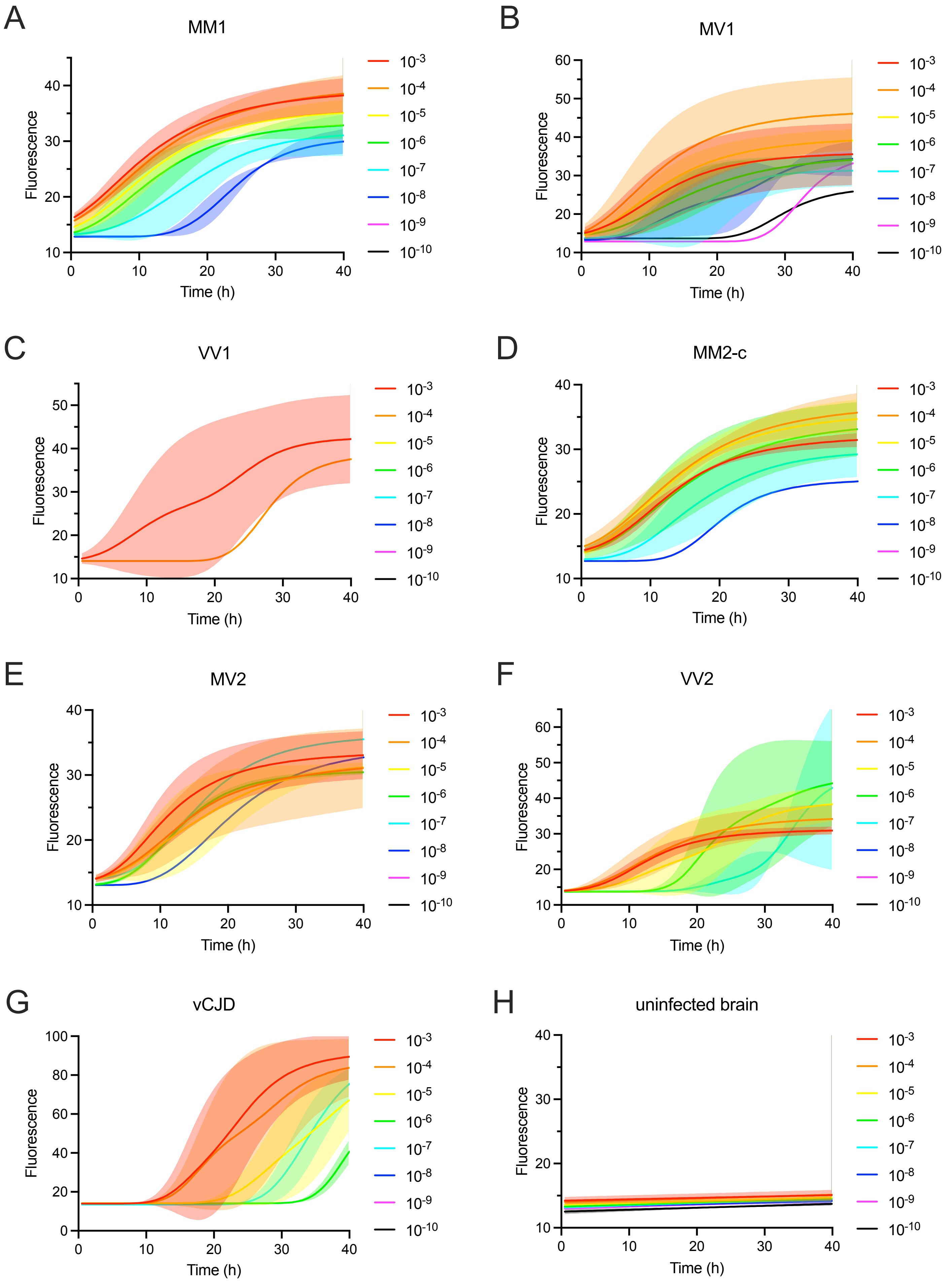

Supplementary Figure 3
